# Supplementary figures and images for: Lifelong absence of microglia alters hippocampal glutamatergic networks but not synapse and spine density
Source: EMBO Rep. 2024 Apr 8;25(5):16. doi: 10.1038/s44319-024-00130-9 (PMC11094096; doi:10.1038/s44319-024-00130-9)

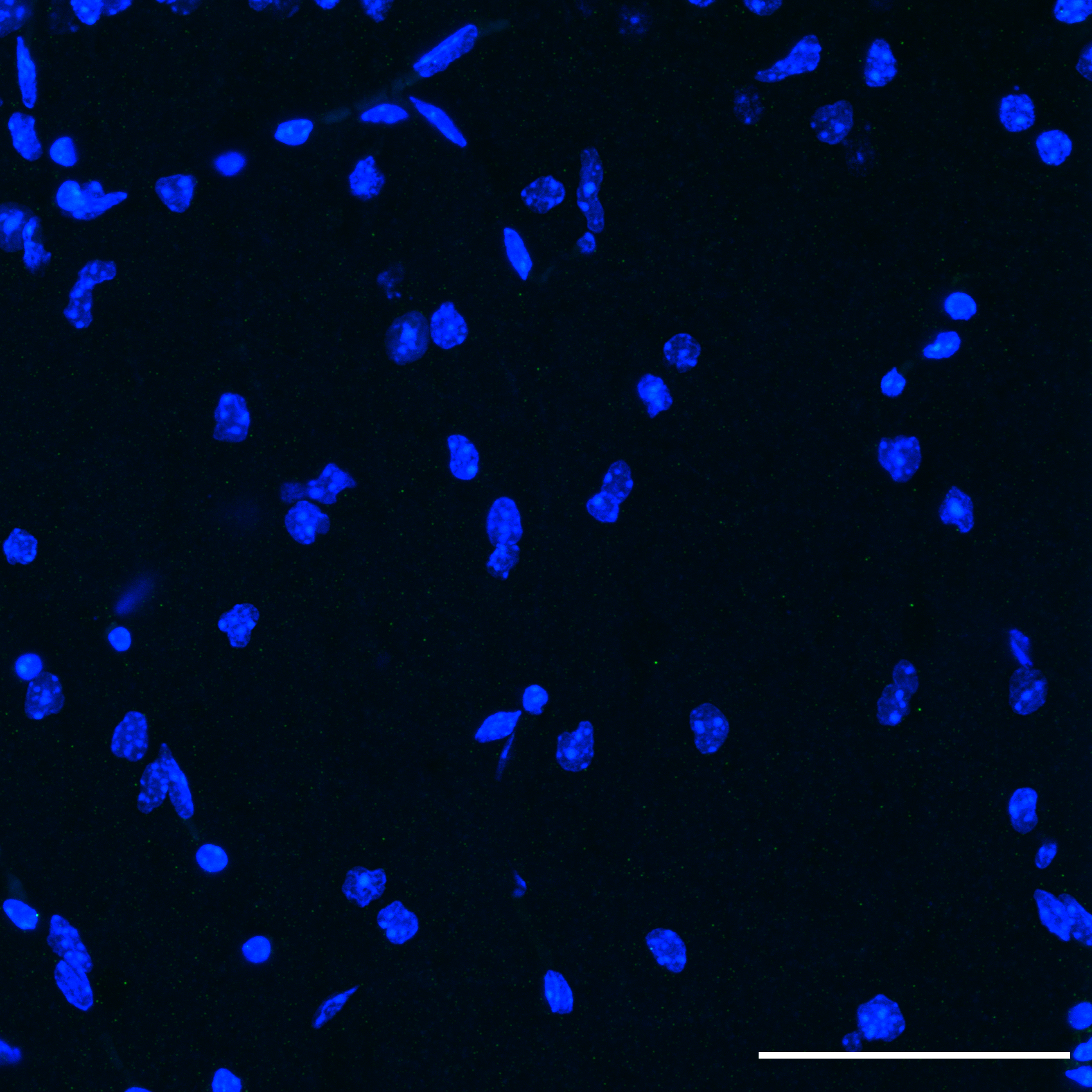

Supplement: Supplementary file 1 — Source data Fig. 1 [file 44319_2024_130_MOESM1_ESM.zip › Figure 1/1A/KO.tif]

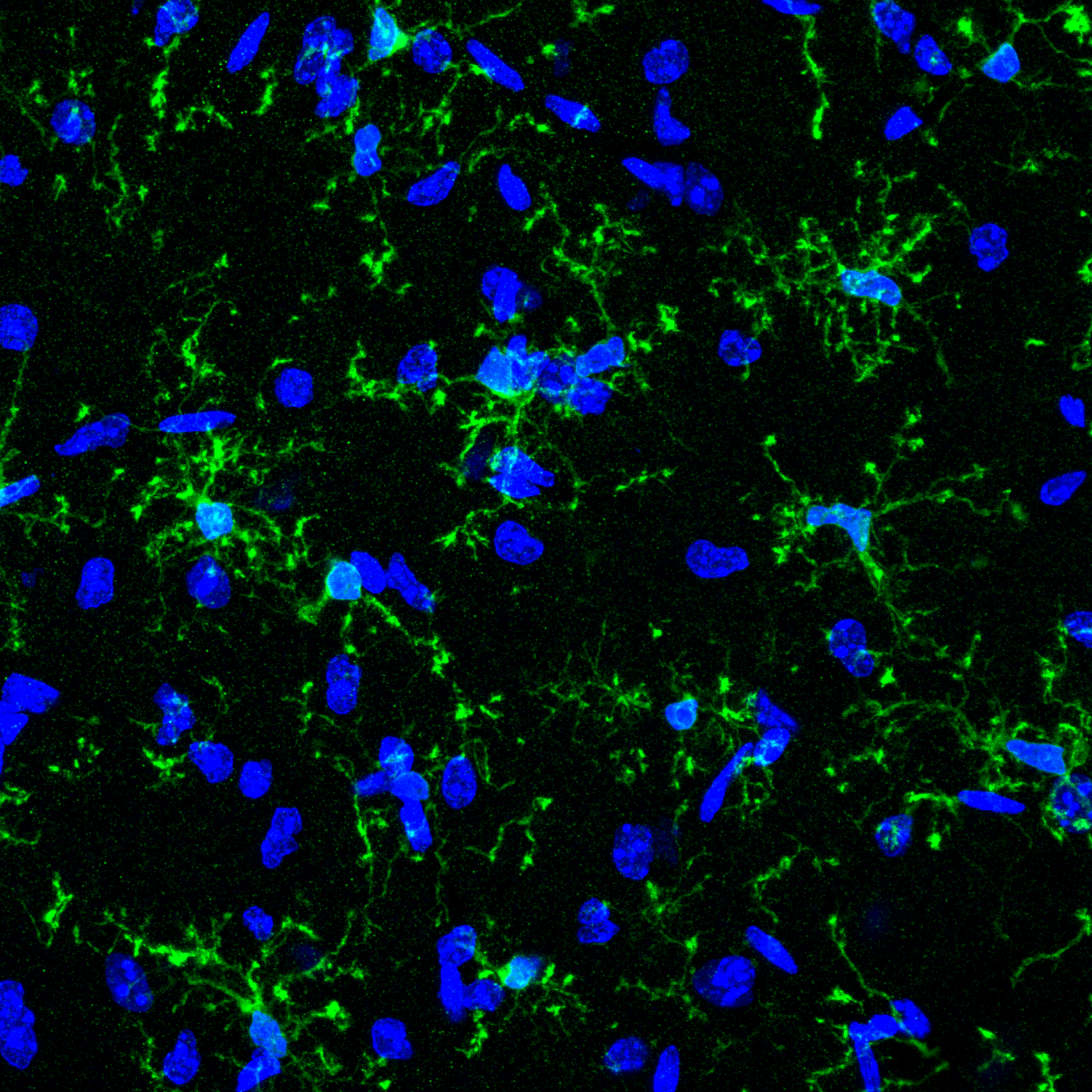

Supplement: Supplementary file 1 — Source data Fig. 1 [file 44319_2024_130_MOESM1_ESM.zip › Figure 1/1A/WT.tif]

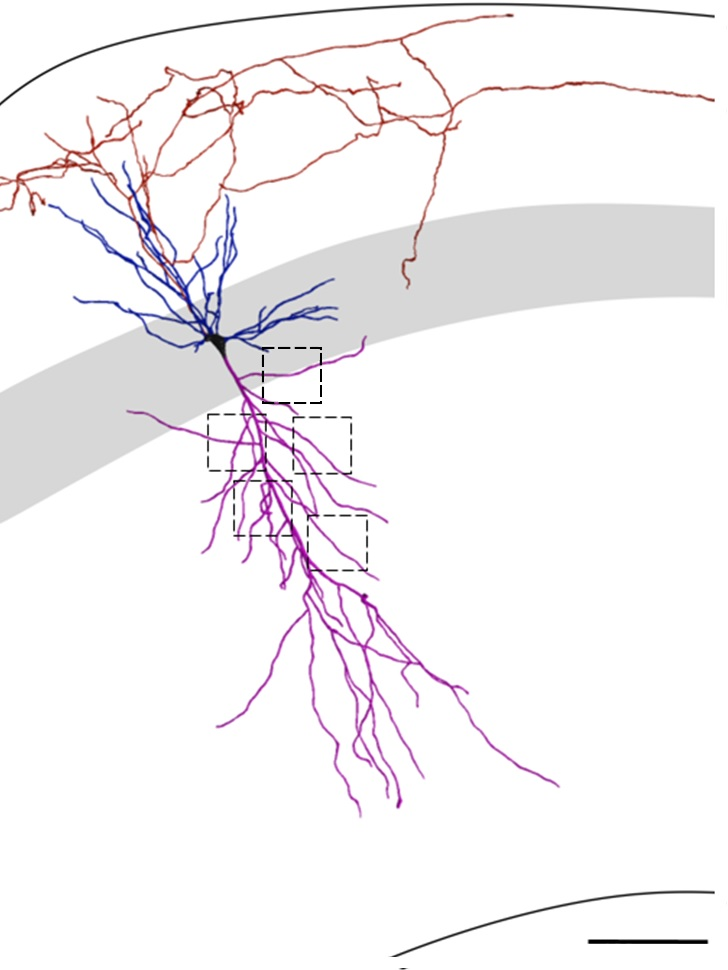

Supplement: Supplementary file 1 — Source data Fig. 1 [file 44319_2024_130_MOESM1_ESM.zip › Figure 1/1B/Fig1B.tif]

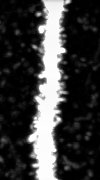

Supplement: Supplementary file 1 — Source data Fig. 1 [file 44319_2024_130_MOESM1_ESM.zip › Figure 1/1C/KO.tif]

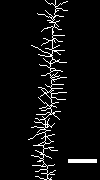

Supplement: Supplementary file 1 — Source data Fig. 1 [file 44319_2024_130_MOESM1_ESM.zip › Figure 1/1C/KO_sk.tif]

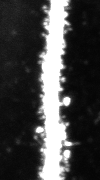

Supplement: Supplementary file 1 — Source data Fig. 1 [file 44319_2024_130_MOESM1_ESM.zip › Figure 1/1C/WT.tif]

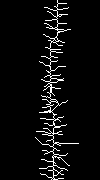

Supplement: Supplementary file 1 — Source data Fig. 1 [file 44319_2024_130_MOESM1_ESM.zip › Figure 1/1C/WT_sk.tif]

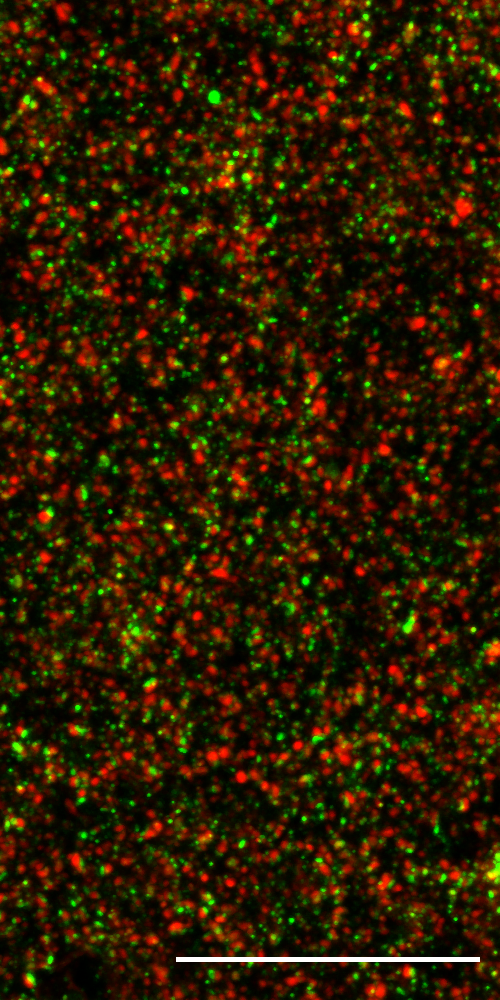

Supplement: Supplementary file 1 — Source data Fig. 1 [file 44319_2024_130_MOESM1_ESM.zip › Figure 1/1J/KO_merge_scale.tif]

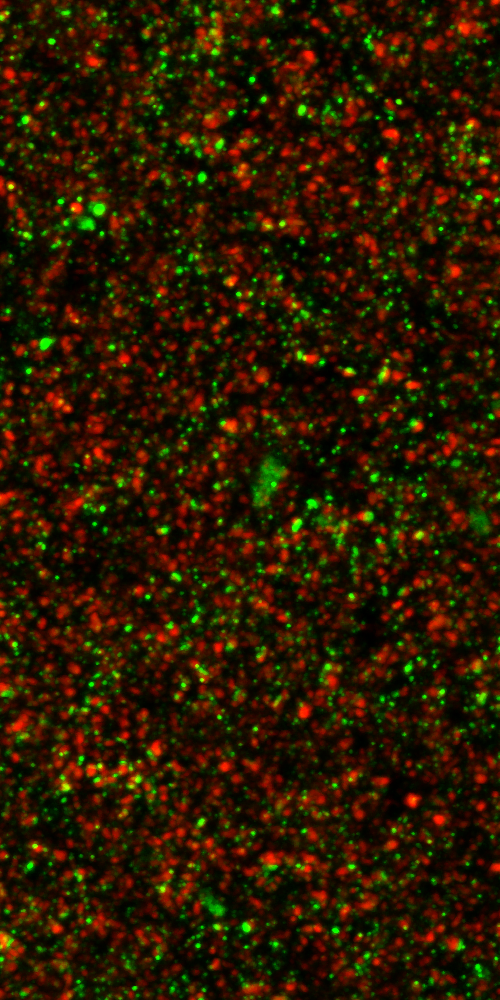

Supplement: Supplementary file 1 — Source data Fig. 1 [file 44319_2024_130_MOESM1_ESM.zip › Figure 1/1J/WT_merge.tif]

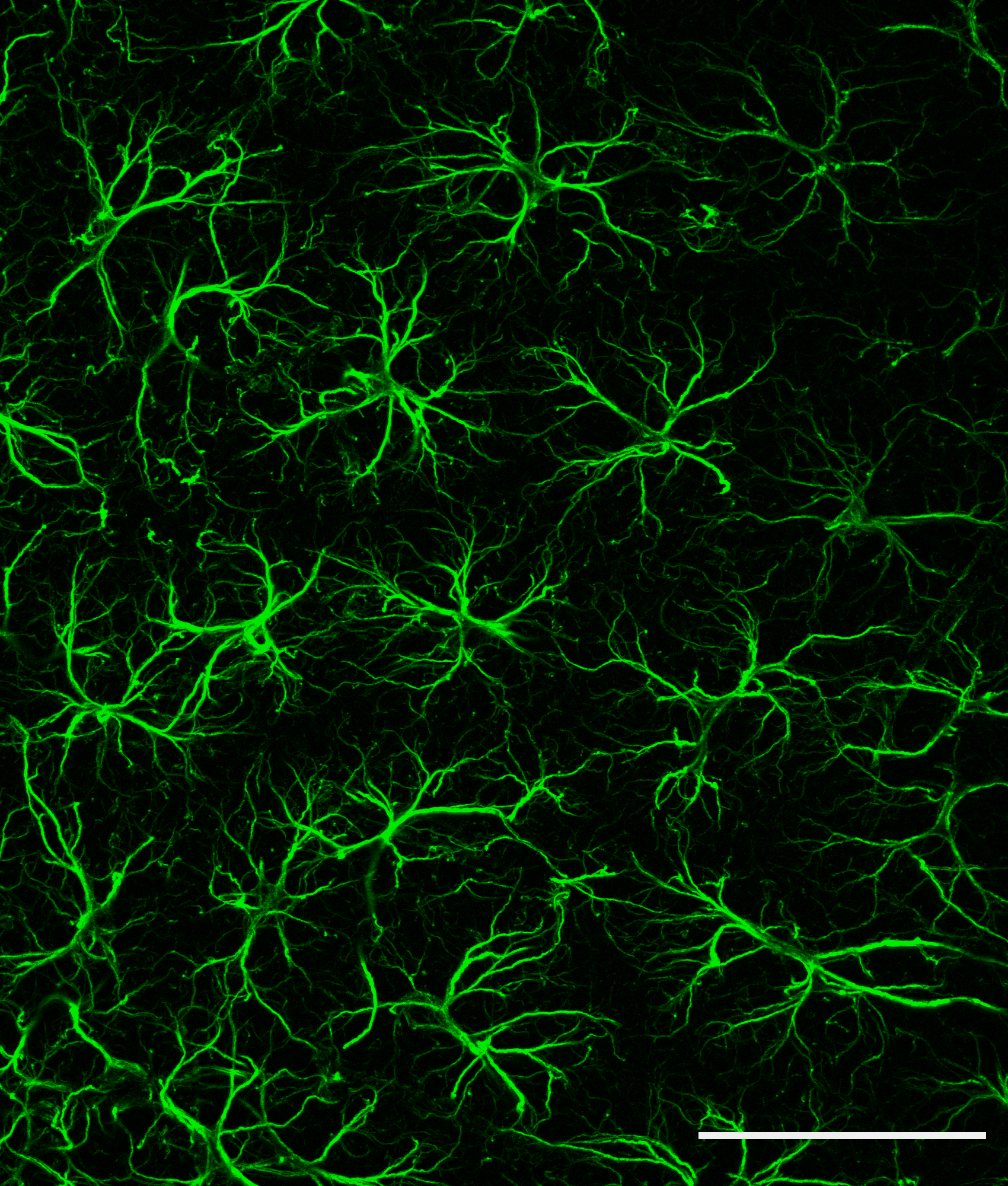

Supplement: Supplementary file 7 — Source data Fig. 7 [file 44319_2024_130_MOESM7_ESM.zip › Figure 7/7A/KO_GFAP.tif]

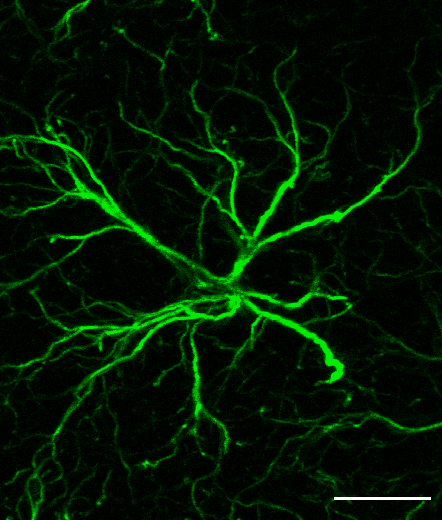

Supplement: Supplementary file 7 — Source data Fig. 7 [file 44319_2024_130_MOESM7_ESM.zip › Figure 7/7A/KO_GFAP_single.tif]

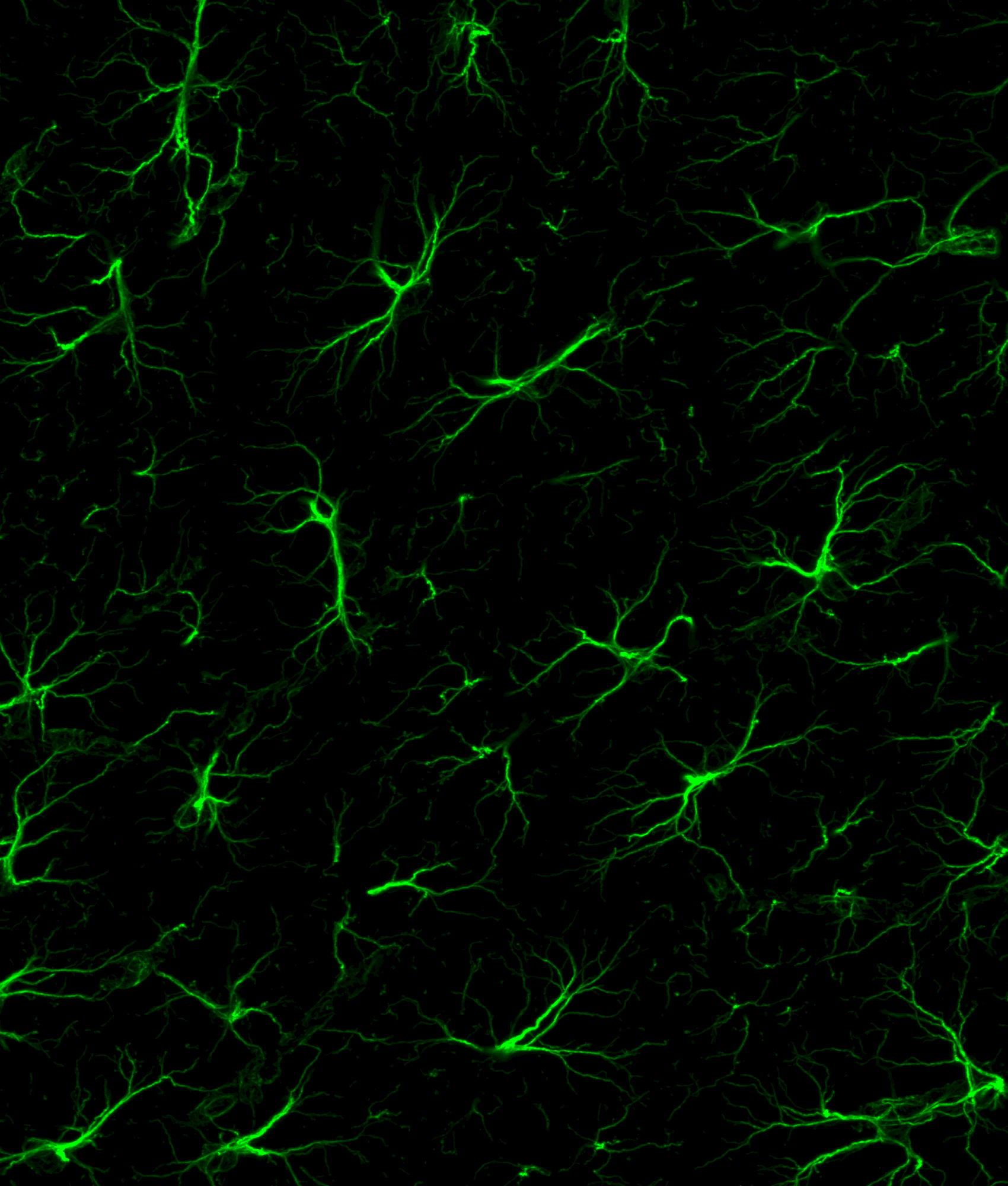

Supplement: Supplementary file 7 — Source data Fig. 7 [file 44319_2024_130_MOESM7_ESM.zip › Figure 7/7A/WT_GFAP.tif]

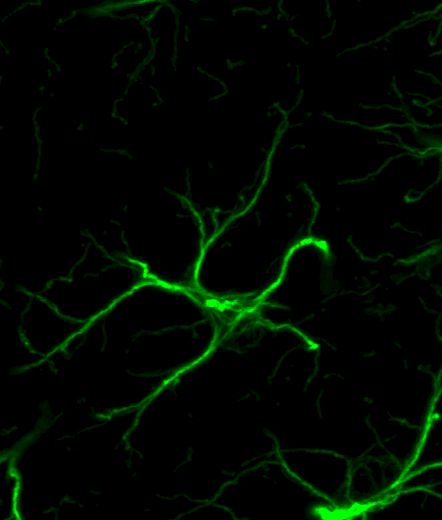

Supplement: Supplementary file 7 — Source data Fig. 7 [file 44319_2024_130_MOESM7_ESM.zip › Figure 7/7A/WT_GFAP_single.tif]

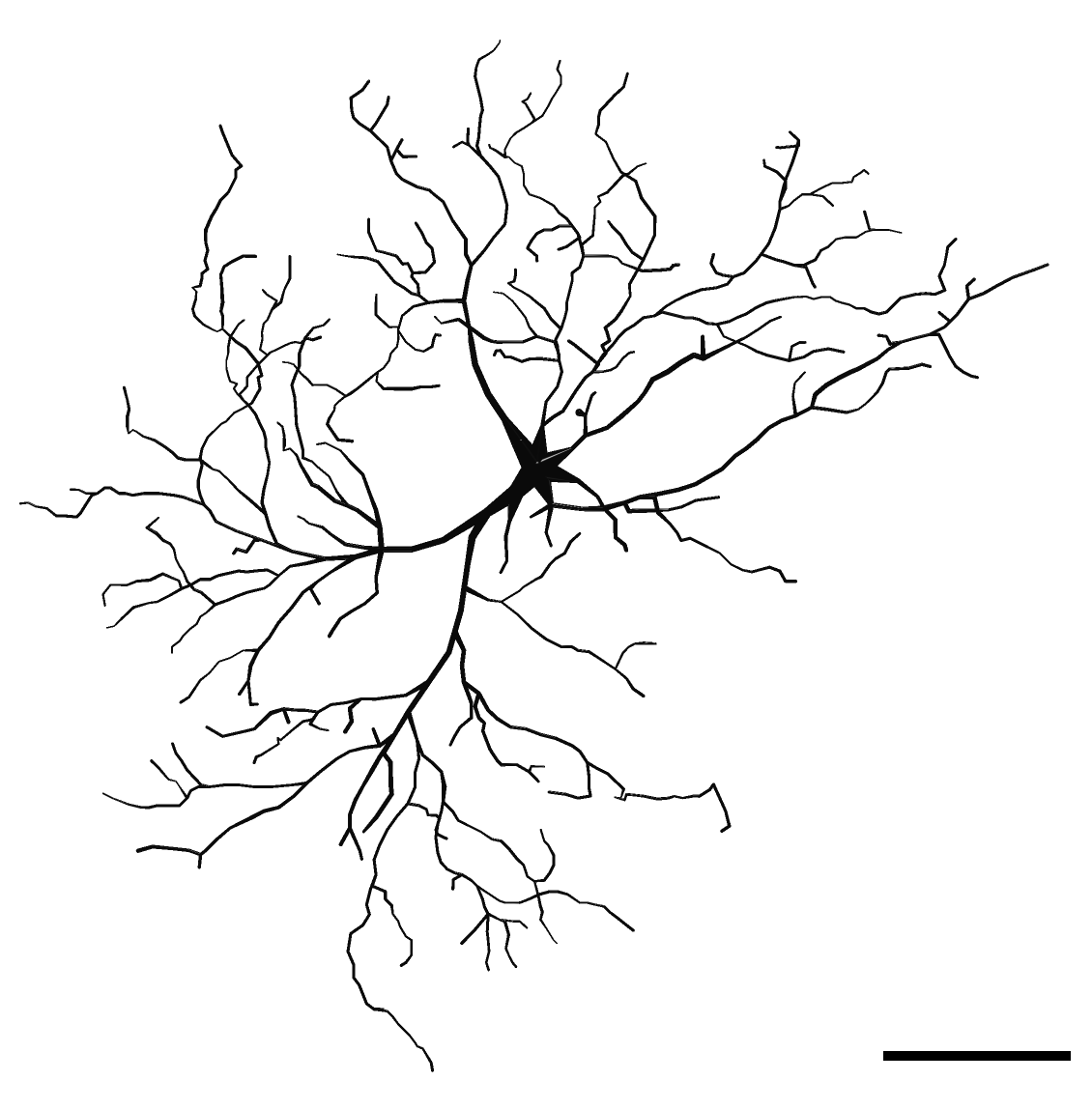

Supplement: Supplementary file 7 — Source data Fig. 7 [file 44319_2024_130_MOESM7_ESM.zip › Figure 7/7E/KO_sk.tif]

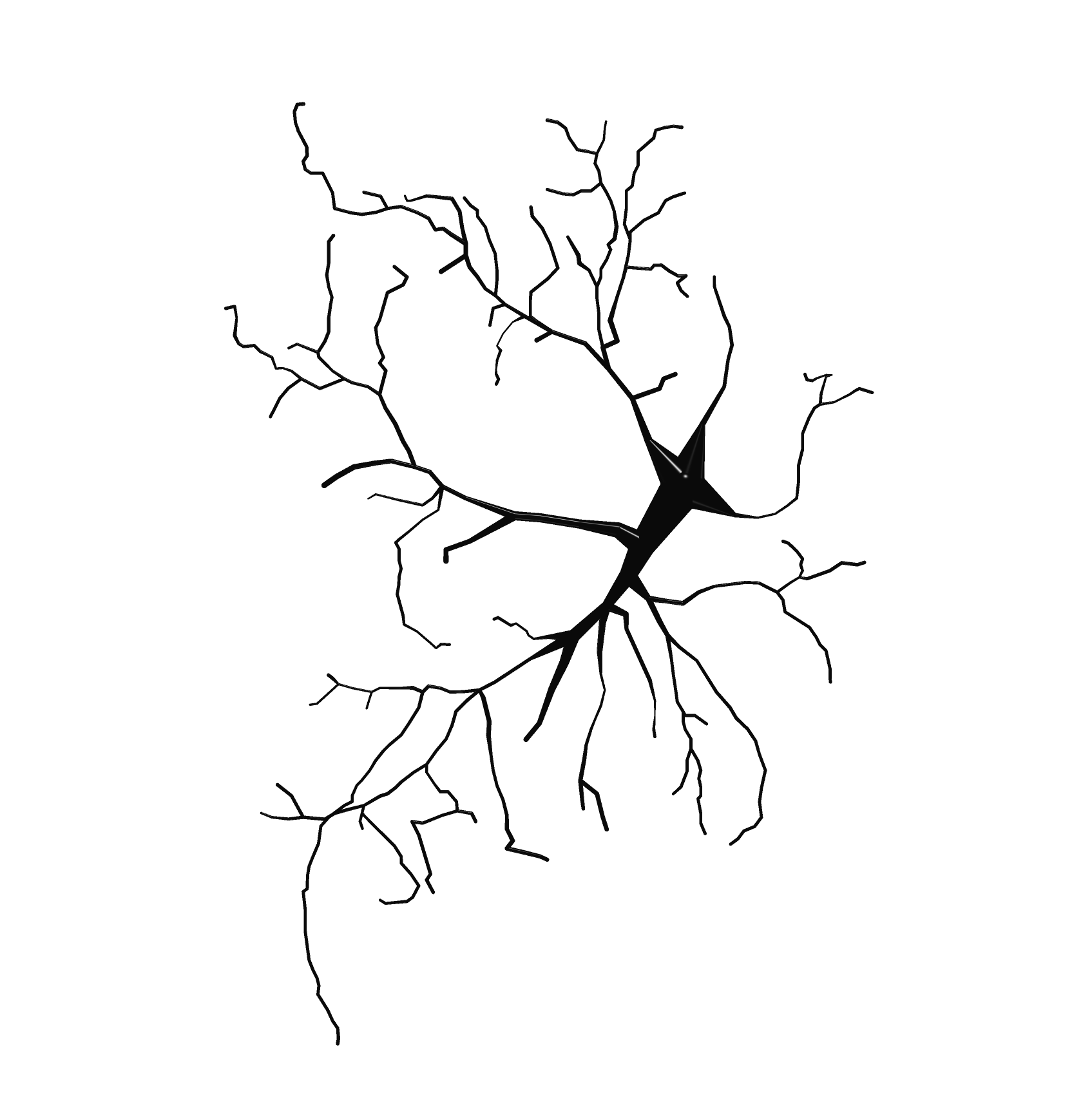

Supplement: Supplementary file 7 — Source data Fig. 7 [file 44319_2024_130_MOESM7_ESM.zip › Figure 7/7E/WT_sk.tif]

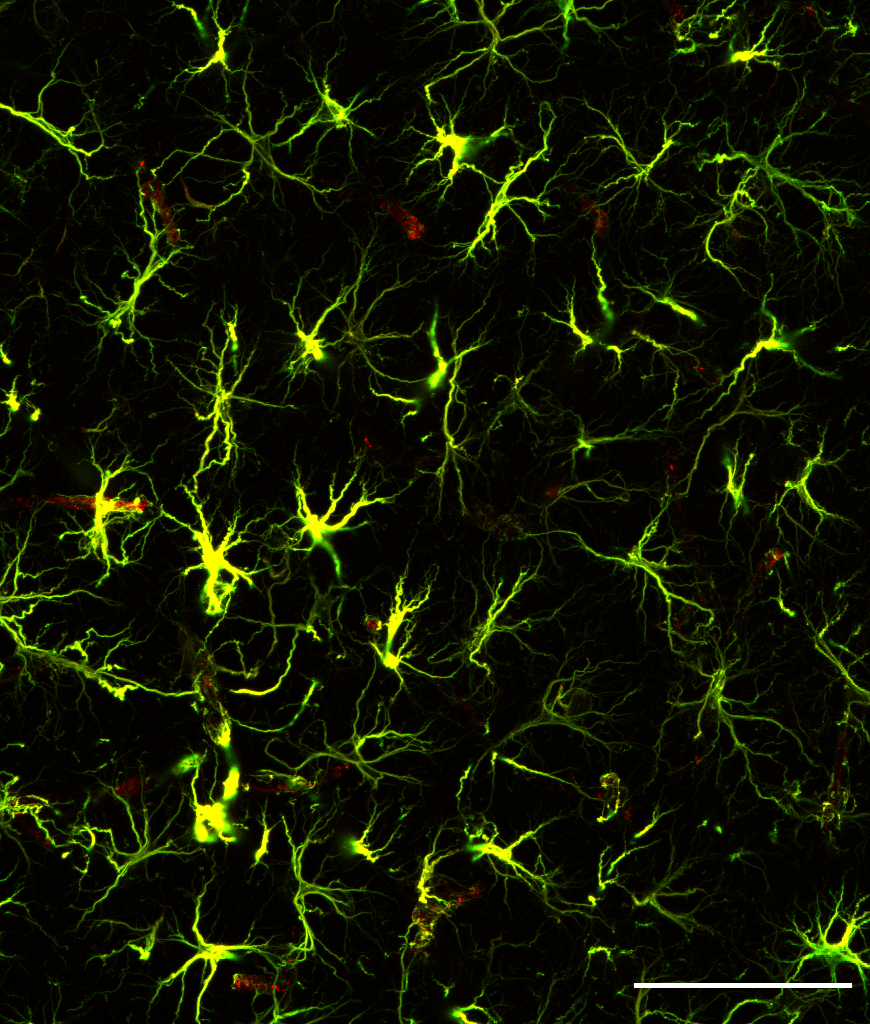

Supplement: Supplementary file 7 — Source data Fig. 7 [file 44319_2024_130_MOESM7_ESM.zip › Figure 7/7I/KO_Merge.lif - 1.tif]

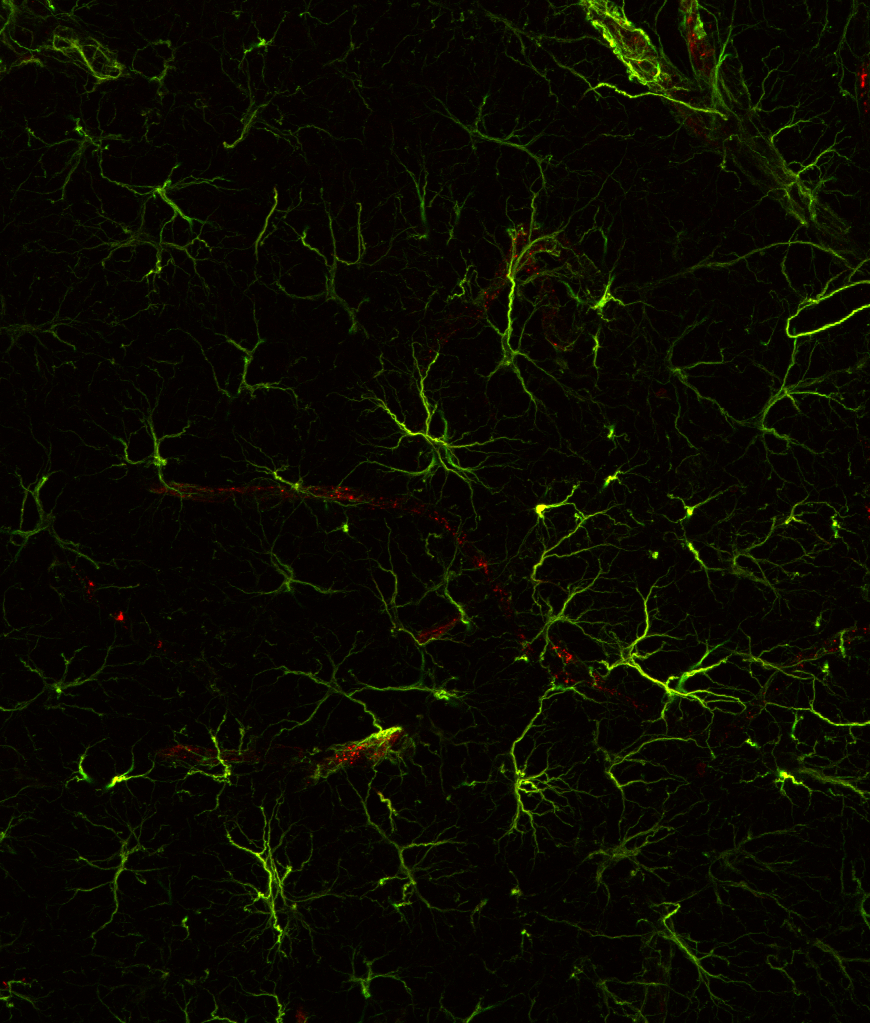

Supplement: Supplementary file 7 — Source data Fig. 7 [file 44319_2024_130_MOESM7_ESM.zip › Figure 7/7I/WT_Merge.lif - 1.tif]

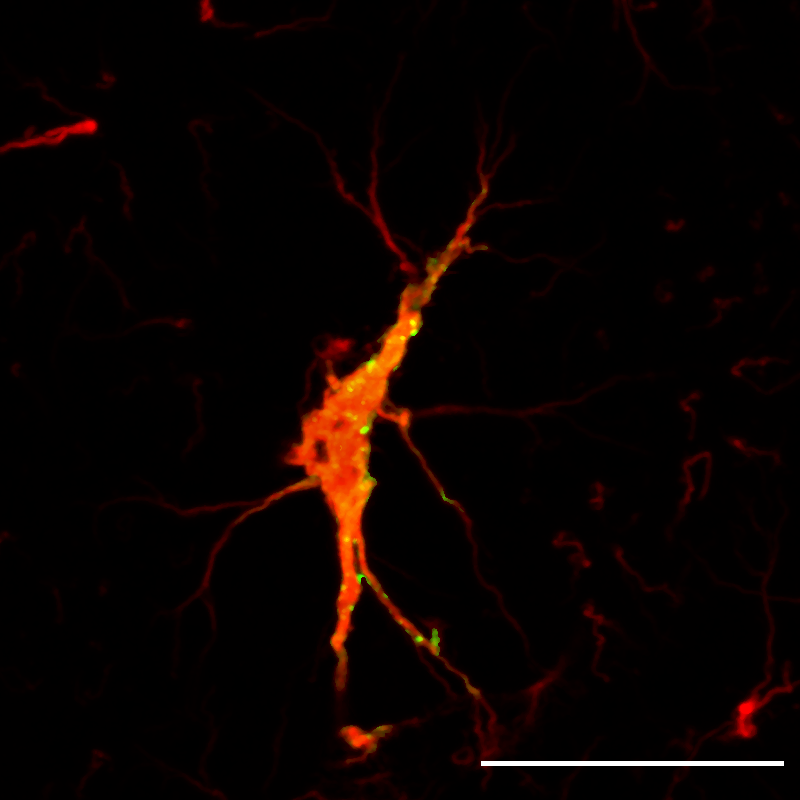

Supplement: Supplementary file 7 — Source data Fig. 7 [file 44319_2024_130_MOESM7_ESM.zip › Figure 7/7N/KO.tif]

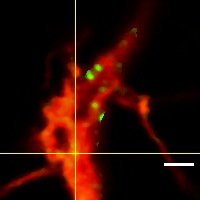

Supplement: Supplementary file 7 — Source data Fig. 7 [file 44319_2024_130_MOESM7_ESM.zip › Figure 7/7N/KO_Homer1_Ortho1.tif]

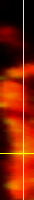

Supplement: Supplementary file 7 — Source data Fig. 7 [file 44319_2024_130_MOESM7_ESM.zip › Figure 7/7N/KO_Homer1_Ortho2.tif]

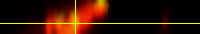

Supplement: Supplementary file 7 — Source data Fig. 7 [file 44319_2024_130_MOESM7_ESM.zip › Figure 7/7N/KO_Homer1_Ortho3.tif]

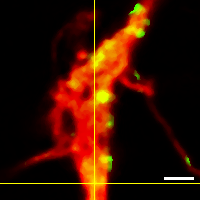

Supplement: Supplementary file 7 — Source data Fig. 7 [file 44319_2024_130_MOESM7_ESM.zip › Figure 7/7N/KO_VGlut1_Ortho1.tif]

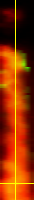

Supplement: Supplementary file 7 — Source data Fig. 7 [file 44319_2024_130_MOESM7_ESM.zip › Figure 7/7N/KO_VGlut1_Ortho2.tif]

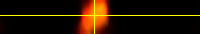

Supplement: Supplementary file 7 — Source data Fig. 7 [file 44319_2024_130_MOESM7_ESM.zip › Figure 7/7N/KO_VGlut1_Ortho3.tif]

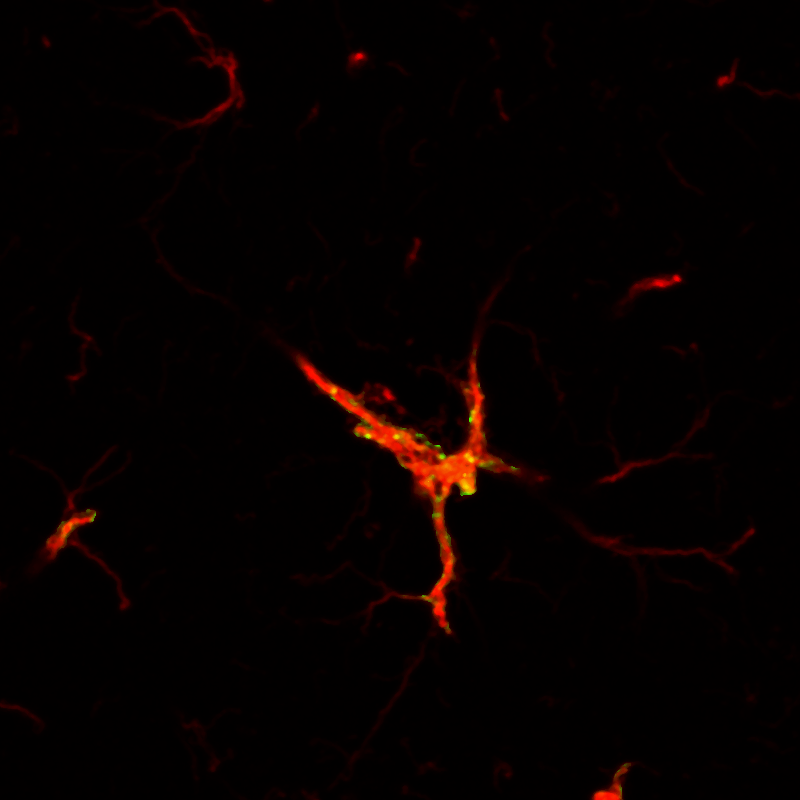

Supplement: Supplementary file 7 — Source data Fig. 7 [file 44319_2024_130_MOESM7_ESM.zip › Figure 7/7N/WT.tif]

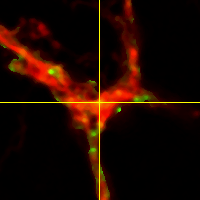

Supplement: Supplementary file 7 — Source data Fig. 7 [file 44319_2024_130_MOESM7_ESM.zip › Figure 7/7N/WT_Homer1_Ortho1.tif]

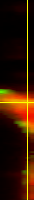

Supplement: Supplementary file 7 — Source data Fig. 7 [file 44319_2024_130_MOESM7_ESM.zip › Figure 7/7N/WT_Homer1_Ortho2.tif]

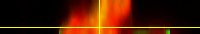

Supplement: Supplementary file 7 — Source data Fig. 7 [file 44319_2024_130_MOESM7_ESM.zip › Figure 7/7N/WT_Homer1_Ortho3.tif]

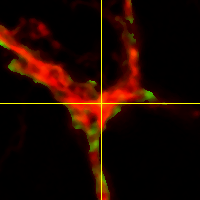

Supplement: Supplementary file 7 — Source data Fig. 7 [file 44319_2024_130_MOESM7_ESM.zip › Figure 7/7N/WT_VGlut1_Ortho1.tif]

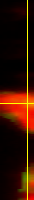

Supplement: Supplementary file 7 — Source data Fig. 7 [file 44319_2024_130_MOESM7_ESM.zip › Figure 7/7N/WT_VGlut1_Ortho2.tif]

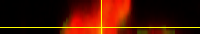

Supplement: Supplementary file 7 — Source data Fig. 7 [file 44319_2024_130_MOESM7_ESM.zip › Figure 7/7N/WT_VGlut1_Ortho3.tif]
