# Supplementary material for: Lifelong absence of microglia alters hippocampal glutamatergic networks but not synapse and spine density
Source: EMBO Rep. 2024 Apr 8;25(5):16. doi: 10.1038/s44319-024-00130-9 (PMC11094096; doi:10.1038/s44319-024-00130-9)
Supplement: Supplementary file 10 — Expanded View Figures [file 44319_2024_130_MOESM10_ESM.pdf]

Expanded View Figures

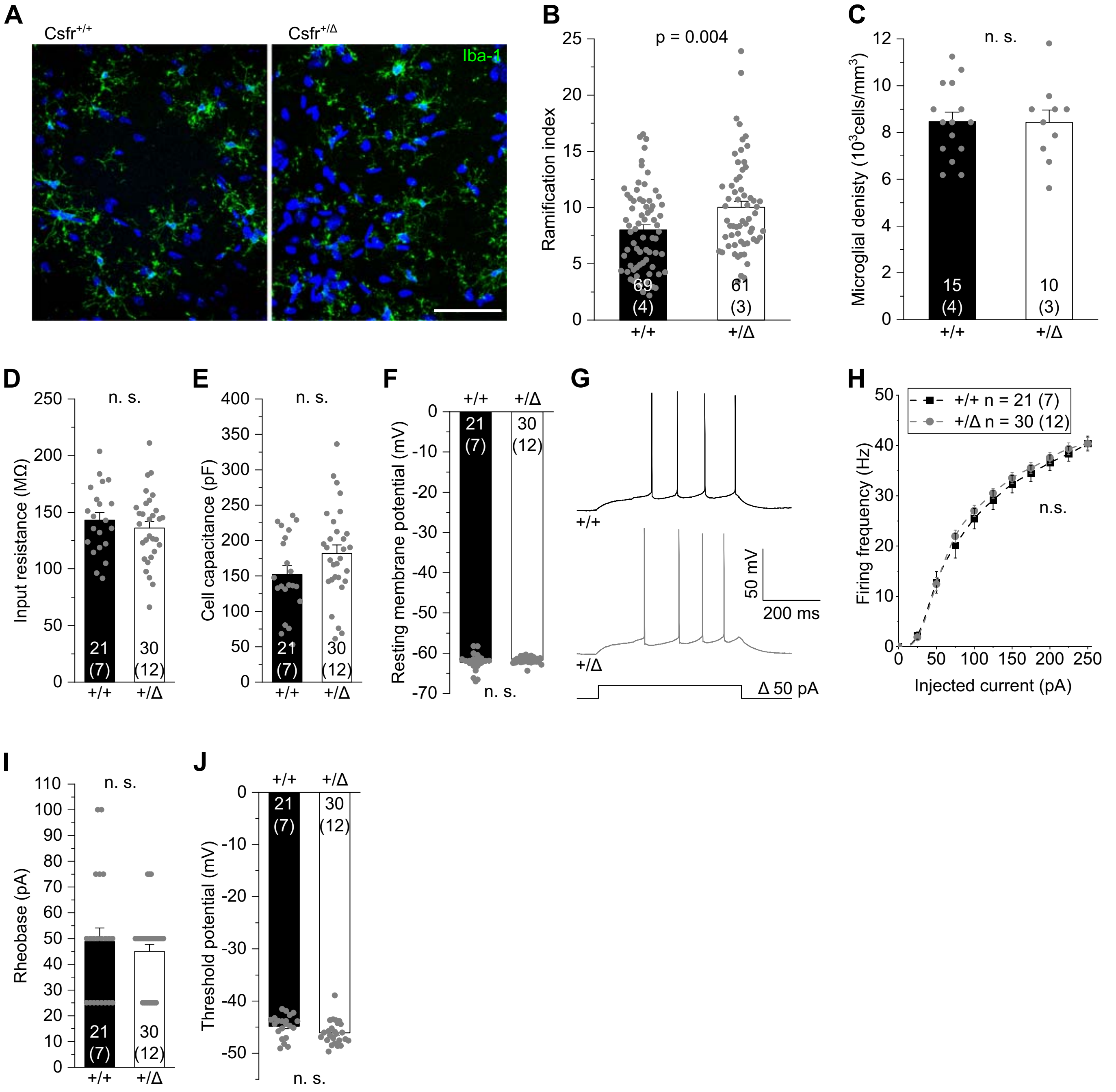

Figure EV1. Normal excitability of CA1 pyramidal cells in heterozygous *Csf1r*$^{+/\Delta FIRE}$ mice.

(A) Specimen confocal images illustrating microglia by Iba1 immunoreactivity (green) in acute hippocampal slices of *Csf1r*$^{+/\Delta FIRE}$ (+/Δ) and WT littermates (+/+). DAPI labeling of cellular nuclei in blue. Scale bar, 50 μm. (B, C) Quantification of microglial ramification (B) and cell density (C) in the CA1 *stratum radiatum*. (D–F) Analysis of input resistance (D), cell capacitance (E) and resting membrane potential (F) of CA1 pyramidal cells. (G) Specimen traces showing action potential firing patterns of CA1 pyramidal cells in response to 500 ms depolarizing current injections. (H). Corresponding course of action potential firing frequencies of CA1 pyramidal cells on increasing depolarizations. (I, J) Values for rheobase (I) and action potential threshold voltage (J). Data information: Data indicate mean ± SEM. Numbers on bars show tested cells or number of slices (C) and (number of animals). *P* values are from unpaired Student's *t* (B–F, J) or Mann–Whitney tests (I) and two-way ANOVA (H).

 © The Author(s)

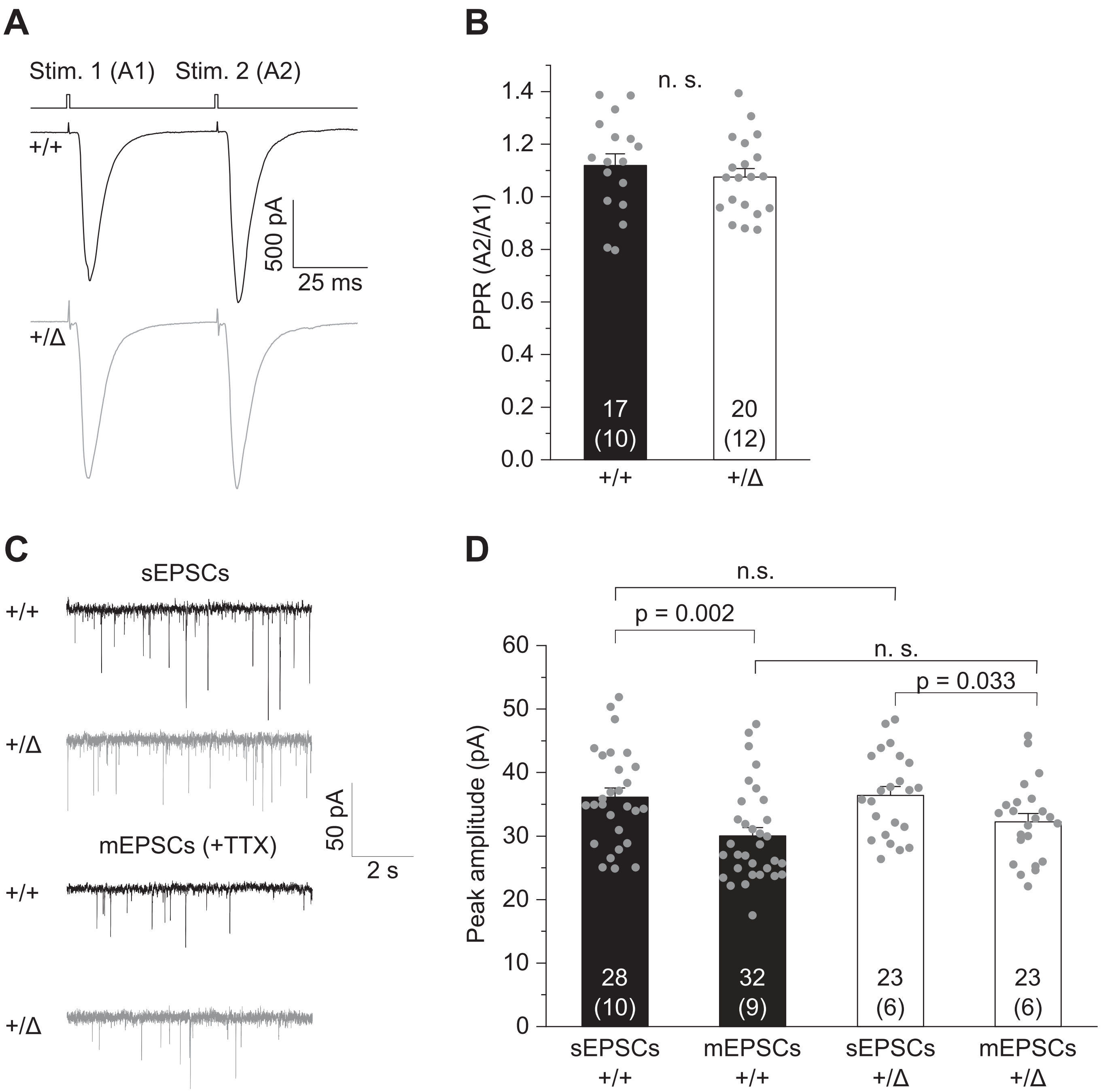

Figure EV2. Normal glutamatergic transmission in heterozygous *Csf1r*$^{+/\Delta FIRE}$ mice.

(**A**) Example traces (EPSCs) of CA1 pyramidal cells after paired-pulse stimulation at 50 ms inter-stimulus intervals. (**B**) Comparison of paired-pulse ratios (PPR) as the quotient of the second vs first EPSC amplitude (A2/A1) of CA1 pyramidal cells. (**C**) Specimen traces showing AMPAR-mediated spontaneous EPSCs (sEPSCs) and miniature EPSCs (mEPSCs) in the presence of 300 nM TTX of CA1 pyramidal cells. (**D**) Comparison of peak amplitudes of sEPSCs and mEPSCs. Data information: Data are represented as mean ± SEM. Numbers on bars indicate tested cells and (number of animals). *P* values are from unpaired Student's *t* (**B**, **D**, for +/Δ and sEPSC comparison) or Mann–Whitney tests (**D**, for +/+ and mEPSC comparison).

© The Author(s)

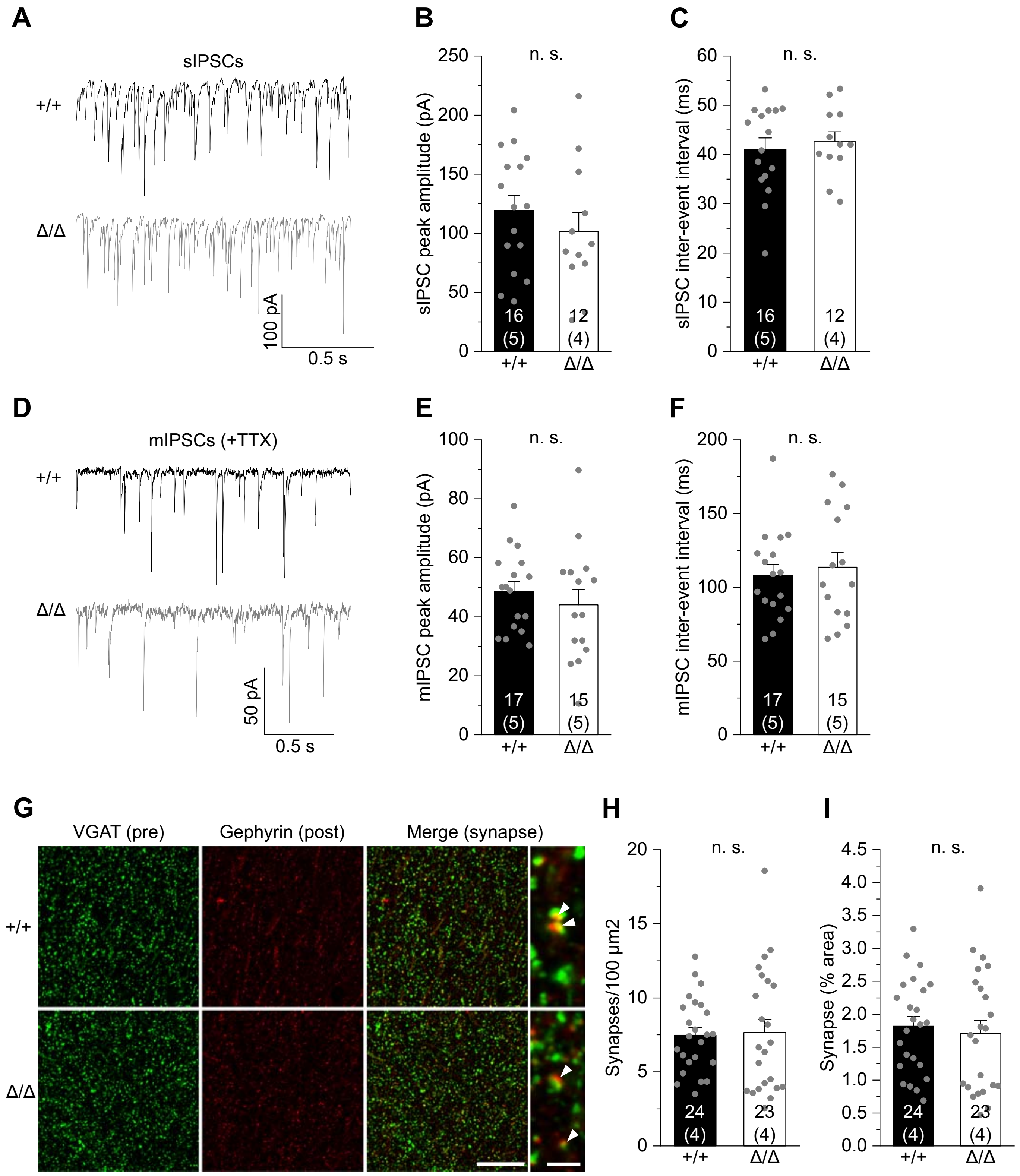
A
sIPSCs
+/+
Δ/Δ
100 pA
0.5 s
B
n. s.
sIPSC peak amplitude (pA)
16
(5)
12
(4)
+/+
Δ/Δ
C
n. s.
sIPSC inter-event interval (ms)
16
(5)
12
(4)
D
mIPSCs (+TTX)
50 pA
0.5 s
E
n. s.
mIPSC peak amplitude (pA)
17
(5)
15
(5)
F
n. s.
mIPSC inter-event interval (ms)
G
VGAT (pre)
Gephyrin (post)
Merge (synapse)
H
n. s.
Synapses/100 µm2
24
(4)
23
(4)
I
n. s.
Synapse (% area)

 © The Author(s)

Figure EV3. Unaltered inhibitory synaptic transmission of CA1 pyramidal cells in *Csf1r*$^{\Delta FIRE/\Delta FIRE}$ mice.

(**A**) Specimen traces showing $GABA_A$ receptor-evoked spontaneous inhibitory postsynaptic currents (sIPSCs) of CA1 pyramidal cells in +/+ and Δ/Δ mice. (**B**, **C**) Comparison of sIPSC peak amplitudes (**B**) and inter-event intervals (**C**). (**D**) Specimen traces showing miniature IPSCs (mIPSCs) of CA1 pyramidal cells. (**E**, **F**) Comparison of peak amplitudes (**E**) and inter-event intervals (**F**). (**G**) Confocal images showing VGAT-labeled presynaptic puncta (green) and Gephyrin-labeled postsynaptic puncta (red) in the CA1 *stratum radiatum* (left and middle) and colocalization of puncta (right, arrowheads). Scale bars, 20 μm and 2 μm (for expanded view). (**H**, **I**) Quantification of colocalized puncta (inhibitory synapses) per 100 $μm^2$ (**H**) and their area covered (**I**). Data information: Data indicate mean ± SEM. Numbers on bars show tested cells and (number of animals). *P* values are from unpaired Student's *t* (**B**, **C**, **E**, **F**, **I**) and Mann–Whitney (**H**) tests.

© The Author(s)

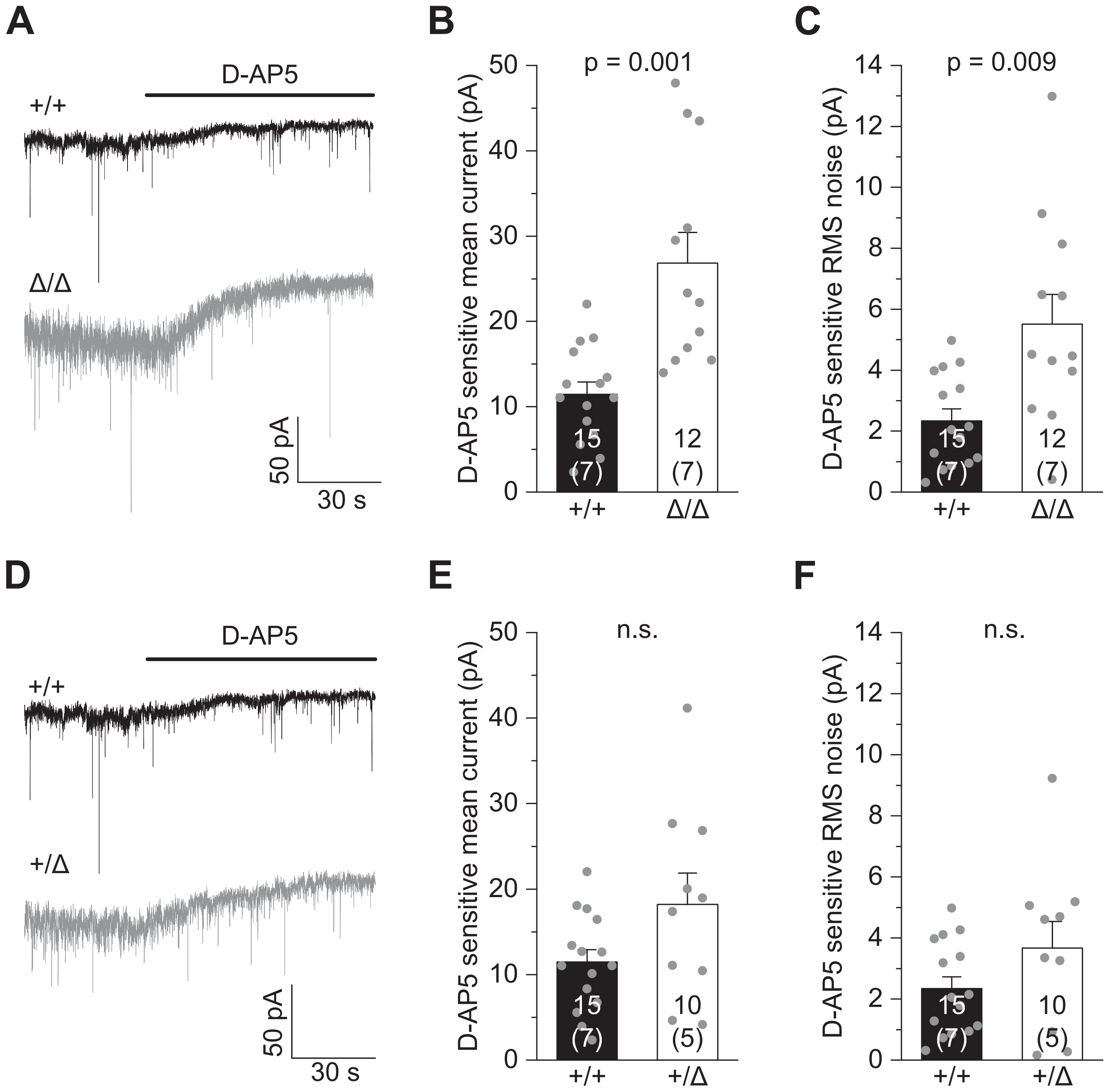

Figure EV4. Increased tonic NMDA current in *Csf1r*$^{\Delta FIRE/\Delta FIRE}$ mice.

(**A**) Example traces showing changes in holding current after application of 50 μM D-AP5 for Δ/Δ compared with +/+ mice, reflecting blockade of all tonic NMDAR-mediated currents in CA1 pyramidal cells. Measurements were done in nominally Mg^{2+}-free extracellular solution in the presence of 300 nM TTX and 10 μM glycine. (**B, C**) Comparison of the D-AP5-sensitive mean holding current (**B**) and root mean square (RMS) noise (**C**) for the different genotypes. Note the contribution of both synaptic and extrasynaptic NMDA receptors to these parameters. (**D–F**) Same as for (**A–C**), but comparison of wild-type (+/+) and heterozygous phenotype (+/Δ). Data information: Data are represented as mean ± SEM. Numbers on bars indicate tested cells and (number of animals). *P* values are from unpaired Student's *t* tests.

 © The Author(s)

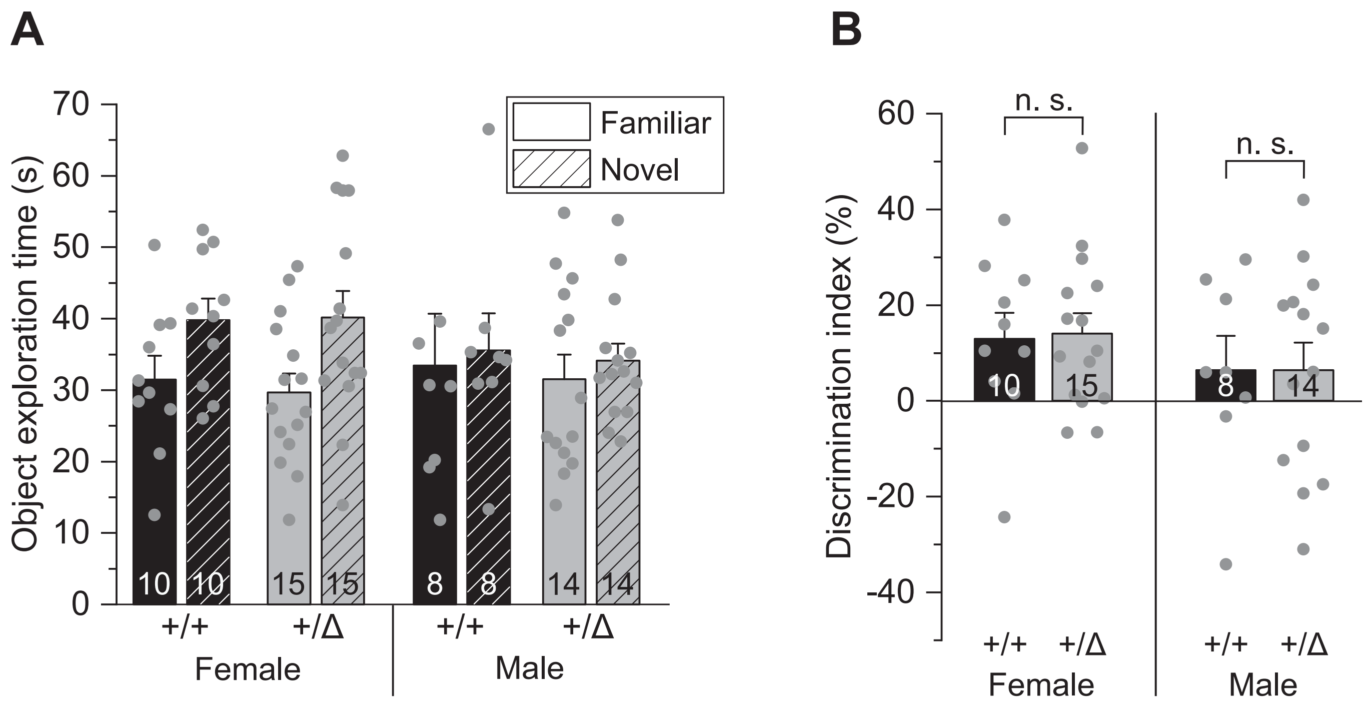

Figure EV5. Unaltered object recognition memory in heterozygous *Csf1r*$^{+/\Delta FIRE}$ mice.

(**A**) Comparison of exploration times of the familiar and novel object after habituation for male and female +/Δ and +/+ mice. (**B**) Comparison of the respective discrimination indices (see "Methods"). Data information: Data are represented as mean ± SEM. Numbers on bars indicate number of tested animals. *P* values are from two-way ANOVA (**B**).
